# Lower circulating levels of chemokine CXCL10 in *Helicobacter pylori*-infected patients with peptic ulcer: Influence of the bacterial virulence factor CagA

**Published:** 2013-03

**Authors:** Abdollah Jafarzadeh, Maryam Nemati, Mohammad Taghi Rezayati, Hossain Khoramdel, Mansooreh Nabizadeh, Gholamhossain Hassanshahi, Hamid Abdollahi

**Affiliations:** 1 Department of Microbiology and Immunology, Medical School, Rafsanjan University of Medical Sciences, Rafsanjan, Iran; 2 Immunology Research Center, Rafsanjan University of Medical Sciences, Rafsanjan, Iran; 3 Molecular Medicine Research Center, Rafsanjan University of Medical Sciences, Rafsanjan, Iran; 4 Department of Microbiology and Immunology, Medical School, Kerman University of Medical Sciences, Kerman, Iran

**Keywords:** *Helicobacter pylori*, Peptic Ulcer, Chemokine CXCL10, Anti-*CagA*, Rafsanjan, Kerman Province, Iran

## Abstract

**Background and Objectives:**

Alterations in CXCL10 (a Th1 chemokine) expression have been associated with various diseases. The aim of this study was to evaluate the serum CXCL10 levels in *H. pylori*-infected patients with peptic ulcer (PU), *H. pylori*-infected asymptomatic (AS) subjects and healthy *H. pylori*-negative subjects, and also to determine its association with bacterial virulence factor *cytotoxin-associated* gene A (*CagA*).

**Materials and Methods:**

Serum samples from 90 *H. pylori* infected patients with PU (70 were anti-*CagA* ^+^, 20 were anti-*CagA* ^-^), 65 AS carriers (40 were anti-*CagA* ^+^, 25 were anti-*CagA* ^-^) and 30 healthy *H. pylori*-negative subjects (as a control group) were tested for concentrations of CXCL10 by using the ELISA method.

**Results:**

The mean serum levels of CXCL10 in PU patients (96.64 ± 20.85 pg/mL) were significantly lower than those observed in AS subjects (162.16 ± 53.31 pg/mL, *P* < 0.01) and the control group (193.93 ± 42.14 pg/mL, *P* < 0.02). In the PU group, the serum levels of CXCL10 in anti-*CagA* ^+^ subjects was significantly higher in comparison to anti-*CagA* ^-^ patients (P < 0.04).

**Conclusion:**

These results showed that the mean concentrations of CXCL10 in *H. pylori*-infected-PU patients was lower than AS carriers and control group. In the PU group, the serum levels of CXCL10 were associated with bacterial factor *CagA*.

## INTRODUCTION

Gastric colonization with *H. pylori* causes peptic ulcer in 10 to 20 percent and gastric cancer in less than 3 percent of those infected (1). The reciprocal interactions between *H. pylori* virulence factors and host genetic background should be consider with regard to the outcome of infection (2). Gastric colonization with *H. pylori* is followed by mucosa recruitment of leukocytes including neutrophils, eosinophils, macrophages, T and B lymphocytes (3). Helper T (Th)-cell associated immune responses in particular, play an important role during *H. pylori* infection regarding the protection or pathologic reactions (4). Th cells were divided principally into Th1 and Th2 subsets which were characterized by a special cytokine profile. Th1 cells secrete cytokines, including interferon (IFN)-γ and IL-2 which lead the activation of macrophages, opsonization and cytotoxicity. In contrast, Th2 cells produce IL-4, IL-10 and IL-13 (5, 6). It has been reported that Th2-related type cytokines including IL-4 and IL-10 prevent Th1 responses, whereas IFN-γ (Th1 cytokine) inhibits Th2 development (5, 6). The exact role of Th1 and Th2 cells in the *H. pylori* infection has not been well known. The results of some investigations have demonstrated that the gastric *H. pylori* specific Th1-mediated immune response contributes in the pathogenesis of *H. pylori*-associated disorders (7). Accordingly, the suppressing of the Th1 responses has been demonstrated to be protective against the development of *H. pylori*-induced pathogenesis (7, 8). Moreover, *H. pylori*- related gastric inflammation has been also associated by enhanced production of Th1-related cytokines including IL-12, IL-18, TNF-alpha, and IFN-γ (9). Controversy, there are some reports regarding the protective effects of Th1 responses to *H. pylori*, so that the reduced gastric Th1- and/or enhanced Th2- related immune responses against *H. pylori* have been associated with the *H. pylori*-related diseases (4, 10).

It should be noted that *H. pylori* strains were divided into *CagA*-positive and *CagA*-negative strains and it has been demonstrated that the *CagA* ^+^ strains were associated with more serious inflammatory reactions and an higher risk of adverse clinical outcomes in western countries (11). The prevalence of the *CagA* ^+^ strains differs among different countries (12). We have previously reported that the seroprevalence of *CagA* ^*+*^ strains in *H. pylori*-infected asymptomatic Iranian adults and children was 72.8% and 67.4%, respectively (13). A strong association also observed between peptic ulcer (PU) disease and infection with *CagA* ^+^ strain (14). Moreover, we have also observed higher serum levels of inflammatory cytokines including IL-17 and IL-18 in *H pylori*-infected individuals, especially in those infected with *CagA* ^+^ strains (15, 16). Furthermore, higher levels of IL-8 have been associated with *CagA* ^+^ strains as compared with *CagA*-negative strain (17).

CXCL10 also called interferon-γ-inducible protein 10 (IP-10) was originally identified as a proinflammatory chemokine mediating leukocyte trafficking, especially contributing to the selective recruitment of Th1 cells in sites of inflammation (18). During inflammatory process chemokine CXCL10 is secreted from neutrophils, eosinophils, monocytes, keratinocytes, epithelial and endothelial cells in response to IFN-γ (6). CXCL10 binds to its receptor, CXCR3, which is mainly expresses on Th1 cells. These CXCR3 expressing cells migrate towards sites of high CXCL10 levels. Therefore, CXCL10 contribute to the selective recruitment of Th1 cells in sites of inflammation (19).

Alterations in CXCL10 expression levels have been associated with inflammatory diseases including infectious diseases (including viral, bacterial, fungal and parasitical infections), autoimmunity and tumor development, indicating an important role of this chemokine in pathogenesis of these diseases (6). CXCL10 is also recognized as a biomarker that predicts severity of various diseases (6, 20). Although, there are many studies on the levels of cytokines (such as IL-1, IL-6, IL-8, IL-10, IL-12, IL-13, IL-17 and IL-18, IL-23, IL-27, TNF-α and IFN-γ) and chemokines (such as CXCL8, CXCL13, CCL19, CCL20, CCL21) in *H. pylori*-infected patients with PU (9, 21–24), however, there is no data on the serum levels of CXCL10. The relationship of chemokine CXCL10 with bacterial virulence factor *CagA* is not also clarified. Moreover, because Th1 cells are thought to play a central role in the protection or pathogenesis of gastric inflammation in *H. pylori* infection, this study focused on the chemokine CXCL10 that is important for the selective recruitment of Th1 cells. This study conducted to evaluate the serum CXCL10 levels in *H. pylori*-infected PU patients [including patients duodenal ulcers (DU) or with gastric ulcer (GU)], *H. pylori*-infected asymptomatic carriers and to compare the levels of CXCL10 between subjects who infected with *CagA* ^+^ or *CagA* ^-^ strains to clarify possible association.

## MATERIALS AND METHODS

### Subjects

Overall, 90 *H. pylori*-infected PU patients (age: 38.72 ± 12.62 years), 65 *H. pylori*-infected asymptomatic (AS) carriers (age: 38.36 ± 11.99 years) and 30 healthy uninfected (*H. pylori*-negative) subjects (age: 37.90 ± 10.48 years) were enrolled into the study.

In PU patients, the presence of disease verified by upper gastrointestinal endoscopy and none of the patients receiving medicinal treatment (including nonsteroidal anti-inflammatory drugs) at the time of investigation. In PU patients, the *H. pylori* status was determined by rapid urease test (RUT) and serological testing for the existence of serum immunoglobulin G (IgG) to *H. pylori* by using commercial enzyme-linked immunosorbent assay (ELISA) kits. RUT was performed on a biopsy specimen that was obtained during endoscopic examination. The patients were considered positive for *H. pylori* infection if both tests (RUT and serological *H. pylori*-specific IgG) simultaneously and together were positive.

AS and non-infected control groups did not undergo endoscopy and were basically health, with no acute or chronic sickness. In deed, the classification of AS and non-infected control groups has been done only according to the H. pylori infection status.

According to the serological screening of anti-*H. pylori*-specific IgG antibodies, the AS carriers were positive for *H. pylori* infection. The AS carriers as well as the non-infected healthy subjects were recruited among blood donors of Rafsanjan Blood Transfusion Center (Rafsanjan, Kerman province, Iran) and interviewed regarding to gastrointestinal manifestations (e.g., dyspepsia), and none of them had any history of gastrointestinal or any other relevant disorders. Moreover, individuals with past medical history of cardiovascular disease, any suspected immunological disorders, infections, allergy and asthma, thyroid disorders, hypertension, diabetes mellitus, pulmonary disease, renal failure, anemia, neoplasia and use of any drug excluded from the study. Other exclusion criteria were surgery and major trauma within 6 months prior to blood collection. A peripheral blood sample was taken from all participants and the sera were separated and stored at 20°C until analysis.

This investigation evaluated and approved by the Ethical Committee of Rafsanjan University of Medical Sciences. Moreover, patients were included if they agreed for blood sampling.

### Determination of *H. pylori*-specific antibodies

The presence of the serum anti-*H. pylori* IgG were determined by using the commercial enzyme-linked immunosorbent assay (Trinity Biotec, Ireland). According to manufacturer guideline the results were expressed as Immune Status Ratio (ISR) and the values of ≥ 1.1 were considered as positive. The specificity and the sensitivity of the assay was reported to be > 98% by manufacturer. The serum anti-*CagA* IgG antibodies levels were also determined ELISA method by using commercial kit (Diagnostic Bioprobes, Italy). The serum concentrations of anti-*CagA* antibodies were expressed as arbitrary units per milliliter (Uarb/ml) because there is no International Standard Unit. According to the manufacturer's guidelines, the value of 5 Uarb/ml was considered to distinguish the negative samples from positive ones.

### Cytokine assay

The serum concentrations of chemokine CXCL10 were quantified by sandwich ELISA using commercial kits (R & D Systems, Minneapolis, USA). The serum levels of chemokine were quantitated by using standard samples with known concentrations of chemokine and expressed as pg/ml, provided by the manufacturer.

### Statistical analysis

Differences in variables were analyzed by using Student t, ANOVA, Mann-Whitney U, Kruskal-Wallis and Welch tests as appropriate and P values of less than 0.05 were considered significant. All the available data were analyzed by a computer program (SPSS version 18, Chicago, IL, USA).

## RESULTS

The mean serum levels of CXCL10 in PU, AS and uninfected control groups has been demonstrated in Table 1 and Table 2. Statistical analyses by using the ANOVA test demonstrated that the difference of the mean serum CXCL10 levels between PU, AS and uninfected control groups were significant (P < 0.02). The mean serum levels of CXCL10 in PU group (96.64 ± 20.85 pg/mL) was significantly lower than those observed in *H. pylori*-infected AS group (185.69 ± 31.04 pg/mL; P < 0.01; by using t-test) and healthy uninfected control group (193.93 ± 42.14 pg/mL; P<0.02; by using t-test). No significant difference was observed for the mean serum levels of CXCL10 between AS group and uninfected control group. The mean serum levels of CXCL10 in total *H. pylori-*infected subjects (PU patients plus AS subjects) was also significantly lower in comparison to uninfected control group (133.98 ± 18.07 vs 193.93 ± 42.14; P < 0.01; by using Mann-Whitney U test) (Table 2).

**Table 1:** Comparison of the serum CXCL10 levels in peptic ulcer (PU), asymptomatic (AS) and control groups according to gender.

Group	Gender	No.	Serum levels of CXCL10 (pg/mL)
PU	Male	45	94.22 ± 29.98†
	Female	45	99.06 ± 29.34
	Total	90	96.64 ± 20.85
AS	Male	32	174.00 ± 36.46
	Female	33	197.03 ± 50.39
	Total	65	185.69 ± 31.04
Total infected (PU + AS)	Male	77	127.37 ± 23.45
	Female	78	140.51 ± 27.58
	Total	155	133.98 ± 18.07
Control	Male	16	202.37 ± 65.06
	Female	14	184.28 ± 53.81
	Total	30	193.93 ± 42.14

† The serum levels of CXCL10 expressed as mean ± SEM.

**Table 2:** Comparison of the serum CXCL10 levels in patients with peptic ulcer (PU), asymptomatic (AS) and control groups.

Group	Anti-*CagA* status	No.	Serum levels of CXCL10 (pg/mL)
PU	Anti-*CagA* ^+^	70	109.54 ± 26.63†
	Anti-*CagA* ^-^	20	51.50 ± 3.80
	Total	90	96.64 ± 20.85
AS	Anti-*CagA* ^+^	40	200.40 ± 38.24
	Anti-*CagA* ^-^	25	162.16 ± 53.31
	Total	65	185.69 ± 31.04
Total infected (PU + AS)	Anti-*CagA* ^+^	110	142.58 ± 22.21
	Anti-*CagA* ^-^	45	112.97 ± 30.54
	Total	155	133.98 ± 18.07
Control	--------------	30	193.93 ± 42.14

† The serum levels of CXCL10 expressed as mean ± SEM.

The PU patients divided into two groups as having DU (n = 65) or GU (n = 25). Statistical analyses by using the Kruskal-Wallis test demonstrated that the difference of the mean serum CXCL10 levels between DU, GU, AS and uninfected control groups were significant (P < 0.001). The mean serum levels of CXCL10 in DU patients (100.49 ± 27.87 pg/mL) was significantly lower than those observed in AS subjects and uninfected control group (P < 0.01; by using Mann-Whitney U test). Similarly, the mean serum levels of CXCL10 in GU patients (86.64 ± 20.41 pg/mL) was also significantly lower than that observed in AS carriers or uninfected control group (P < 0.01; by using Mann-Whitney U test). No significant difference was observed for the mean serum levels of CXCL10 between DU and GU groups.

The mean serum CXCL10 levels in PU, AS and non-infected control groups according to gender has been summarized in Table 1. No significant differences were observed between men and women of three groups with respect to the mean serum CXCL10 levels.

The results of the serum concentrations of CXCL10 according to anti-*CagA* status have been also demonstrated in Table 2 & Table 3. Statistical analyses by using the Welch test demonstrated that the mean serum CXCL10 levels in PU patients with positive test for anti-*CagA* antibody was significantly higher in comparison to PU patients with negative test for anti-*CagA* antibody (P < 0.04). Although, in AS group, the mean serum levels of CXCL10 in anti-*CagA* positive subjects was also higher than that observed in anti-*CagA* negative individuals but the difference was not statistically significant. Moreover, in total *H. pylori-*infected subjects (PU patients plus AS subjects) the mean serum levels of CXCL10 in anti-*CagA* positive subjects was higher than that observed in anti-*CagA* negative individuals but the difference was not statistically significant (Table 2).

**Table 3:** Statistical comparison of serum CXCL10 concentration between peptic ulcer (PU) and asymptomatic (AS) groups according to the anti-*CagA* status.

Groups and subgroups		Asymptomatic (AS)		Peptic ulcer (PU)	

		(anti-*CagA* ^-^)	(anti-*CagA* ^+^)	(anti-*CagA* ^-^)	(anti-*CagA* ^+^)
**Uninfected control**		0.63*	0.91	0.008	0.01
**AS**	**(anti-*CagA*^-^)**	-	0.55	0.04	0.05
	**(anti-*CagA*^+^)**	0.55	-	0.008	0.01
**PU**	**(anti-*CagA*^-^)**	0.04	0.008	-	0.04
	**(anti-*CagA*^+^)**	0.05	0.01	0.04	-

* The symbol represents p-values (p-values are equal to assigned numbers)

It should be noted that the mean serum levels of anti-*CagA* antibody in PU patients (30.37 ± 3.61 Uarb/ml) was significantly higher in comparison to AS group (17.10 ± 2.59 Uarb/ml; P < 0.006; by using t-test).

## DISCUSSION

The results of the present study showed the lower serum concentrations of CXCL10 in *H. pylori*infected PU patients (including patients with gastric or duodenal ulcers) in comparison to AS and control groups. It has been also previously demonstrated that *H. pylori* fractions are able to suppressing the *in vitro* CXCL10 production by the gastric epithelium (25). Therefore, lower CXCL10 levels may reflect a weak Th1-related immune response in the gastric mucosa of *H. pylori*-infected patients. Some investigations suggest that a strong Th1-related immune response to *H. pylori* may be necessary for protection and clearance of bacteria (4, 10, 26). It is unclear how protection is performed by Th1-mediated mechanisms to control *H. pylori* infection. It should be noted that Th1 cytokines are also powerful inducers of the humoral immune response which may involve in the protection against *H. pylori* (27).

Diminished production of CXCL10 may cause the weak recruitment of the CD4^+^ Th1 cells into the gastric mucosal of *H. pylori*-infected subjects. Therefore, inadequate Th1-related immunity in *H. pylori* infection may cause to gastritis due to development of infection. It seems to be logic that *H. pylori* might have evolved strategies (such as inhibition of the CXCL10 production) to minimize the migration of Th1 cells to the gastric mucosal. Lower CXCL10 levels as observed in *H. pylori*-infected patients likely forgive a selective advantage to this pathogen for evading the host's immune system.

Controversially, it has been reported that the gastric Th1 response against *H. pylori* contributes to the pathogenesis of *H. pylori*-related diseases (7). Down-regulation of the Th1 responses has been shown to be protective against the *H. pylori*-induced pathologic reactions (7, 8). Gastric inflammation with *H. pylori* has been also associated with increased production of Th1-type cytokines including IL-12, IL-18, TNF-alpha, and IFN-γ (9). Accordingly, it seems that an uncontrolled excessive Th1-related immune response may have a pathogenic role rather than protective effect and play a pivotal role in the induction of mucosal damage. Therefore, it seems that both excess or weak Th1-type responses against *H. pylori* may contribute to the development of *H. pylori*-related diseases. Accordingly, an optimal Th1-related immune response may be necessary for the elimination and/or control of *H. pylori* infection.

The exact molecular mechanisms that are responsible for the suppression of CXCL10 in H. *pylori*-infected PU patients remain to be determined. Previous studies have demonstrated that certain *H. pylori* derivatives can modulate the local and/or systemic immune response (28). Similar mechanisms may also be account for the inhibition of chemokine CXCL10 production.

It should be also mentioned that there was no significant difference between DU and GU groups regarding to the mean serum levels of CXCL10. In both groups the mean serum levels of CXCL10 was significantly lower than those observed in AS and uninfected control group. This finding represents that the similar immunopathologic mechanisms may contribute to the pathogenesis of the DU and GU diseases. The results here also demonstrated that only in PU group the mean serum levels of CXCL10 was significantly lower than AS and control group but the differences of mean serum levels of CXCL10 between AS and control group were not significant. These results represent that *H. pylori* infection solely is not associated with lower CXCL10 levels. Indeed, lower CXCL10 levels is associated with development of PU disease rather than only *H. pylori* infection as seen in AS subjects.

*H. pylori* have several virulence factors such as *CagA* that may influence the clinical outcome of the infection. The results of some investigations have demonstrated that gastric colonization with *CagA* ^+^ strains of *H. pylori* induces more severe mucosal damage in comparison with *CagA*-negative strains (17, 29, 30). However, in other studies the relation of *CagA* ^+^ strains with more severe gastric inflammation has not been observed (31).

The results of the present study showed that in PU group, the mean levels of CXCL10 was significantly higher in subjects who were positive for anti-*CagA* antibody in comparison with individuals with negative test for anti-*CagA* antibody. It has been also reported that the *CagA* can preferentially activate Th1-related responses through inducing IL-12 secretion by macrophages (7). Moreover, higher levels of IL-8, IL-18 and IL-17 have been observed in subjects who infected with *CagA*+ strains of *H. pylori* (15–17). These observations are consistent with our findings. Our results represent a clear association between the infection with *CagA*+ strains of *H. pylori* and the serum levels of chemokine CXCL10. Therefore, the *CagA* or *CagA*-associated factors may directly and/or indirectly trigger the expression of chemokine CXCL10.

The results of some other investigations, however, failed to show any association between the *H. pylori* virulence factors such as *CagA* and the production of cytokines including IL-6, IL-8, IL-10, IL-27 and TNF-α (24, 32–34). The cytokine gene polymorphisms including polymorphisms of the IL-1, TNF-α and IFN-γ have been also related with *H. pylori*-associated gastric adenocarcinoma and PU (35). Furthermore, wide geographical variations have been observed in the genotype of *CagA* ^+^ strains of *H. pylori* (36, 37). Accordingly, both bacterial and host parameters should be considered for understanding the *H. pylori*-associated inflammatory reactions.

### Study limitations

Our study may have several limitations: First, asymptomatic carriers were determined by serological detection of anti-*H. pylori* specific IgG. Since the *H. pylori* have various immunodominant antigens, measurement of the antibodies to some of those antigens has been reported as a reliable marker for detection of *H. pylori* infection (38). It has been reported that the serological tests are valuable non-invasive methods for the diagnosis of infection because they are simple and comfortable. The most frequently used serological test for diagnosis of *H. pylori* infection is ELISA due to its simplicity, low cost, high sensitivity and highly accurate for the detection of *H. pylori* infection in adults (39). We utilize an ELISA method for diagnosis of *H. pylori* infection by using commercial kit. The sensitivity of used ELISA kit in this investigation has been previously showed to be 100% for Iranian population (40). In the present study, in PU patients, the *H. pylori* infection was determined by RUT and the presence of *H. pylori*-specific IgG by using ELISA method. The PU patients considered positive for *H. pylori* infection if both tests were simultaneously positive. However, in AS and non-infected control groups, the *H. pylori* infection was determined according to the presence of *H. pylori*-specific IgG. Because RUT is an invasive method we could not obtain the biopsy sample from AS and control groups due to ethical restrictions. However, it has been reported that the results of serological methods are comparable with biopsy based RUT for diagnosis of *H. pylori* infection (40). Collectively, the estimation of the overall reliability of commercially available kits that measure IgG antibodies for the diagnosis of *H. pylori* infection have showed that serology is an exact technique for diagnosis of *H. pylori* infection (41). According to the manufacturer's guidelines, the sensitivity and specificity of our assay were > 98%. We suppose that the high sensitivity and specificity of the used method in the present study likely exclude most of false reactions. By using the same technique, we have previously reported that the seropositive rate of *H. pylori* infection in healthy Iranian adults and children was 67.5% and 46.6%, respectively (13). Since the *CagA* is immunogenic, detection of the serum IgG antibodies to the *CagA* antigen has been also reported as a reliable parameter of carriage of a *CagA* ^*+*^ *H. pylori* strain (42). Second, measurement of chemokine CXCL10 was performed on samples that were stored at -20°C. We cannot eliminate the possibility of protein degradation. However, this phenomenon should affect both samples of cases and controls in a similar manner. Third, the detection of the mucosal levels of chemokine CXCL10 and the determination of the frequencies of the T cell subsets including CD4^+^ T cells, CD8^+^ T cells, regulatory T cells and Th17 cells in the peripheral blood samples or in the gastric biopsies from PU patients and the association of these parameters with chemokine CXCL10 were not parts of the protocol. Finally, the examining chemokine CXCL10 during the PU disease might introduce the predictive or prognostic values for this chemokine. The results of our investigation encourage more studies in these fields.

In conclusion, the present study indicates that serum levels CXCL10 were decreased in *H. pylori*-infected PU patients including patients with DU or GU. In PU group, the *CagA* virulence factor associated with the serum levels of CXCL10.

### Declaration of interest

The authors declare no conflicts of interest.
